# In Situ Atomic-Scale Observation of Silver Oxidation Triggered by Electron Beam Irradiation

**DOI:** 10.3390/nano11041021

**Published:** 2021-04-16

**Authors:** Hui Zhang, Tao Xu, Yatong Zhu, Wen Wang, Hao Zhang, Dundong Yuan, Litao Sun

**Affiliations:** 1 SEU-FEI Nano-Pico Center, Key Laboratory of MEMS of Ministry of Education, School of Electronic Science and Engineering, Southeast University, Nanjing 210096, China; huiz@seu.edu.cn (H.Z.); 230189517@seu.edu.cn (Y.Z.); 230169400@seu.edu.cn (W.W.); 220191460@seu.edu.cn (H.Z.); 220191420@seu.edu.cn (D.Y.); 2 Center for Advanced Materials and Manufacture, Southeast University–Monash University Joint Research Institute (Suzhou), Suzhou 215123, China

**Keywords:** oxidation, crystal growth, electron beam irradiation, in situ, transmission electron microscopy

## Abstract

Understanding the mechanism of metal oxidation processes is critical for maintaining the desired properties of metals and catalysts, as well as for designing advanced materials. In this work, we investigate the electron beam induced oxidation of silver using in situ transmission electron microscopy. The additions of Ag-O columns on {111} and {110} planes were captured with atomic resolution. Interestingly, oscillatory growth on {110} planes was observed, which resulted from the double effect of electron beam irradiation. It was found that not only thermodynamic factors but also kinetic factors played significant roles in morphology evolutions. These results can facilitate the fundamental understanding of the oxidation process of Ag and provide a promising approach for the fabrication of desired nanostructures.

## 1. Introduction

Due to its superior electrical and thermal conductivity and high reflectivity over a wide optical range, sliver (Ag) is used in a wide variety of applications in space vehicles, such as conductive materials [1], as well as detectors and sensors for monitoring atomic oxygen concentrations [2,3]. Spacecrafts located on low earth orbit (LEO) are exposed to a harsh oxidizing environment [4,5,6], which contains a substantial amount of atomic oxygen (flux: 10^15^ atoms·cm^−2^·s^−1^) [7,8]. Atomic oxygen, generated by the dissociation of molecular oxygen due to ultraviolet radiation, causes an enhanced oxidation of materials on spacecrafts, which severely shortens the life of the spacecrafts [1,9,10]. Therefore, insights on the oxidation processes taking place when atomic oxygen interacts with the materials are necessary for designing long-life spacecrafts in the LEO.

Meanwhile, Ag is a promising material with broad potential applications in catalysis, antibacterial actions, fluorescence enhancement, and so on [11,12,13]. Understanding the atomic-level oxidation processes of Ag is of significant scientific and technological interest. Some laboratory studies of the interaction between Ag and oxygen have been performed and reported as well [14,15,16,17]. An important finding is that Ag is easily oxidized by atomic or ionized oxygen but not by molecular oxygen [5,8]. Sun et al. [18] found that the oxidation of Ag were able to be triggered by the ionized oxygen dissociated by electron beam irradiation, and the new oxide phase forms by the nucleation of new grains adjacent to the Ag. Sheng et al. [14] also showed the reversible oxidation and reduction that can be controlled by changing the dose rate of the electron beam. Although some studies were carried out regarding the oxidation behavior of Ag, a direct interpretation of atomistic processes and kinetics corresponding to Ag is still challenging due to a limitation of spatial resolution in conventional experimental methods.

For this study, applying an aberration-corrected transmission electron microscope (TEM), we study the atomic-scale oxidation process of Ag that is activated by electron beam irradiation at room temperature. The electron beam irradiation directly activates the oxidation process, as the ionization of adsorbed oxygen molecules dissociated by the electron beam plays a major role in the oxidation. Different from previously reports, we found that the oxidation of Ag proceeded by conformal growth through the adatom process. Moreover, the addition of atom columns on {110} planes was observed for the first time, as well as the oscillatory growth of Ag_2_O. The Ag_2_O crystals grow via the manner of layer by layer, and the lattice planes with relatively lower surface energies have a faster growth rate. These results provide atomic insight into the oxidation dynamics of Ag.

## 2. Materials and Methods

Ag nanowires with featured structures were well synthesized by a solution-phase method demonstrated by Xia et al. [19,20]. In a typical synthesis, 3 mL ethylene glycol (EG; anhydrous, 99.8%, Aladdin, Shanghai, China) solution (0.1 M) of Ag nitrate (Aladdin, Shanghai, China) and 3 mL EG solution (0.6 M) of poly(vinyl pyrrolidone) (PVP; Aladdin, Shanghai, China) were injected into 5 mL EG using a two-channel syringe pump at a rate of ~0.3 mL·min^−1^. After the injection, the reaction mixture was further heated at 160 °C for 60 min under stirring. Then, the obtained reaction mixture was repeatedly washed with acetone and deionized water (5–10 times by volume) using centrifuge at 2000 rpm for ~20 min. The as-synthesized Ag nanowires were in face-centered cubic (FCC) phase and typically have 5-fold twinned structures with an average diameter of ~70 nm and lengths up to several μm, as shown in Figure S1. The in situ oxidation experiments were conducted in an image aberration-corrected TEM (FEI Titan 80–300 TEM operating at 300 kV, Thermo Fisher Scientific, Waltham, MA, USA).

## 3. Results

### 3.1. In Situ Oxidation Process

**Figure 1:**
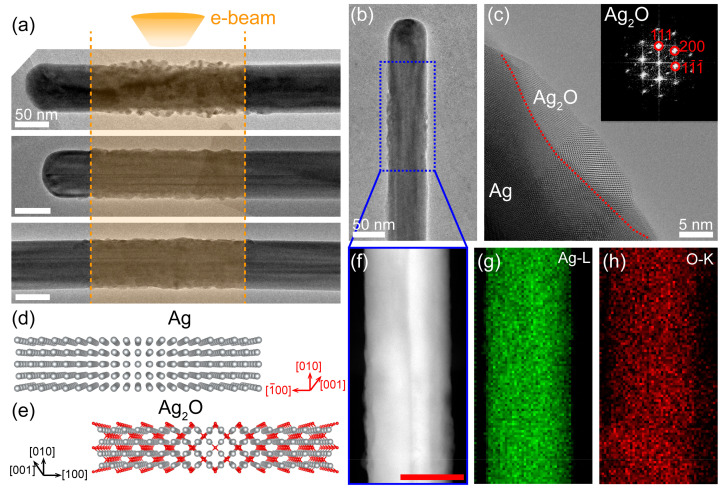
Oxidation of Ag nanowires triggered by electron beam. (**a**) Morphology changes only occur in the regions exposed to the electron beam. (**b**) TEM image of Ag nanowire. The blue dotted lines highlight the oxidation area. (**c**) High resolution TEM image of the oxidation area. The red dotted line shows the interface of Ag and Ag_2_O; the inset is the fast Fourier transform (FFT) pattern of Ag_2_O crystal. (**d**,**e**) Atomic structures of Ag and Ag_2_O, respectively. Red and gray balls represent the O atoms and Ag atoms, respectively. (**f**–**h**) HAADF-STEM image and EDS elemental maps of the oxidation area in (**b**). Scale bars for (**a**,**b**,**f**) at 50 nm, for (**c**) at 5 nm.

As shown in Figure 1, both the morphologies and structures of Ag nanowires underwent an obvious transformation under the electron beam irradiation because the residual oxygen (~3 × 10^−5^ Pa pressure) in the chamber can react with Ag under electron beam bombardment [14]. Obviously, the morphology changes only occur in the regions exposed to the electron beam (Figure 1a), which indicates that electron beam irradiation plays an important role in the oxidation process of Ag. The high-resolution TEM (HRTEM) image and the corresponding fast Fourier transform (FFT) pattern (Figure 1c), and the energy dispersive spectroscopy (EDS) mappings (Figure 1g,h) confirm that the reaction product is Ag_2_O (JCPDS File No. 76-1393). It is expected that the morphology changes of Ag nanowires result from the volume expansion Ag_2_O (Figure 1d,e).

**Figure 2:**
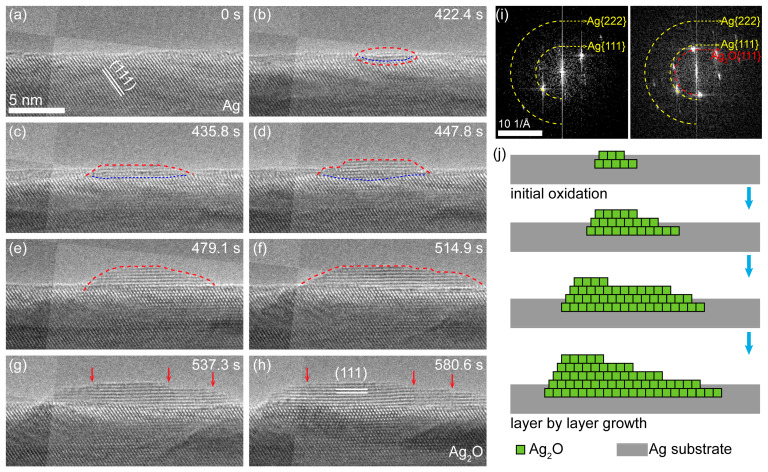
Layer-by-layer growth of Ag_2_O on the Ag nanowire surface. (**a**–**h**) Time sequenced HRTEM images showing the growth process of Ag_2_O grain. The red arrows show the step edges of the layer, and blue dashed lines indicate the interface between Ag and Ag_2_O. Dose rate at 10,700 e·Å^−2^·s^−1^. (**i**) Corresponding FFT pattern analysis indicating the Ag_2_O formation during the surface oxidation. (**j**) Schematic diagram showing the layer-by-layer growth of Ag_2_O grain.

To further investigate the process of the oxidation, a series of HRTEM images were acquired, as shown in Figure 2 (see Video S1 for more details). The original surface of Ag nanowire is shown in Figure 2a, in which the white lines illustrate the {111} lattice plane of Ag (2.35 Å). A new Ag_2_O nanocrystal nucleated on the subsurface of the Ag nanowire after the electron beam irradiation for several minutes (Figure 2b). Further growth involved the lateral extension and the longitudinal thickening of the oxide layer. The formation of Ag_2_O nanocrystals can also be verified from the FFT patterns in Figure 2i, where new spots of 2.73 Å (it should be assigned to the {111} planes of Ag_2_O) appeared after electron beam irradiation.

The growth rate of lateral extension was much higher than that of the longitudinal, which indicates that the growth of Ag_2_O crystal proceeds in the mode of “layer by layer”. The observations are consistent with the previous reports in [21,22], where Ag_2_O crystal are always bonded on the {111} faces due to the low surface free energy [23]. Specifically, the growth may proceed as follows (Figure 2j). Firstly, free ionized oxygen is captured by Ag atoms at defect sites and could further bond with another Ag atom on the surface. When the Ag-O-Ag groups are formed side by side, more Ag atoms can be captured to form a small Ag_2_O crystal nucleus. As the reaction continues, the step edge site of the Ag_2_O crystallite bonds with more and more Ag and O atoms, and a complete oxide layer appears on the surface of Ag nanowire. It should be noted that the migration of a Ag/Ag_2_O interface to Ag substrates is observed, which indicates that the diffusion of O species through the oxide layer and the reaction at the interface also contribute to the growth of Ag_2_O.

Different from the study by Sun et al. [18], the oxidation process of Ag nanowires observed in this work was carried out through the adatom process rather than the conventional solid-solid transformation mechanism. In other words, the oxidation of Ag proceeds through conformal growth rather than nucleation of new grains adjacent to the Ag specimen.

### 3.2. Mass Transport in Crystal Growth

The layer-by-layer growth of Ag_2_O requires the transportation of Ag atoms to the specific site on the outer surface, which can be achieved by surface and/or bulk diffusion. Thus, the diffusion of Ag atoms seems to be a limiting factor in the growth of the oxide layer.

**Figure 3:**
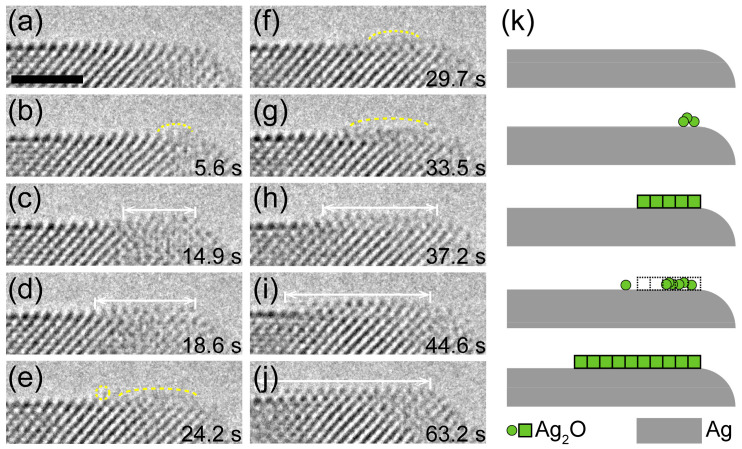
The formation of new layer on the Ag_2_O substrate. (**a**–**j**) Time-resolved HRTEM images showing the formation of a new Ag_2_O layer on a flat Ag_2_O (110) surface. Dose rate at 9600 e·Å^−2^·s^−1^. An amorphous layer (labeled by yellow dashed line) emerges on the surface (**b**) and then expands laterally with a clear lattice (**c**–**d**). The extensional layer transforms between amorphous and crystalline and eventually leads to the formation of a larger layer. (**k**) Scheme of the formation of new Ag_2_O layer growth process. Scale bar at 2 nm.

Thermodynamically speaking, surface diffusion is energetically preferred as a result of the lower energy barrier. Figure 3a–j show an atomic event that a new Ag_2_O layer forms at the right side of the as-formed oxide substrate. In 5.6 s, an amorphous layer emerged on the right side of the Ag_2_O substrate surface. After that, several Ag atom columns were successively added to the neighboring sites, resulting in the formation of a narrow Ag_2_O layer. These extension layers frequently nucleated at the edge site of the oxide substrate, which indicates that the Ag atoms may be transported to the top surface of oxide substrate by surface diffusion. During the initial growth of Ag_2_O, Ag-O columns were bonded to the as-formed Ag_2_O in an amorphous state. Under the electron beam irradiation, the disordered and ordered states of the oxide alternated with each other until a stable Ag_2_O crystal layer was formed (Figure 3k). It should be noted that the growth of an oxide layer is not only controlled by thermodynamic factors but also by kinetic factors. Kinetically, it is possible that Ag atoms migrate to the top surface of the oxide by bulk diffusion [24]. However, the atomic events representing bulk diffusion in the experiments were not captured due to the limitation of instrument. Here, we want to point out that both surface diffusion and bulk diffusion routes play important roles in the formation of oxide layer, but the surface diffusion may be preferred due to the low diffusion barrier.

### 3.3. Layer-by-Layer Growth on {110} Planes

**Figure 4:**
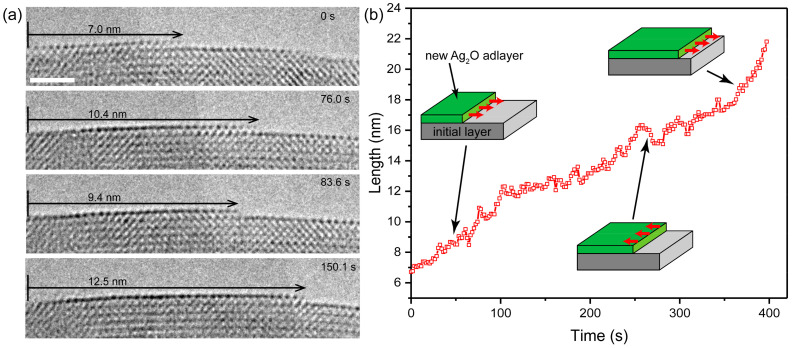
Oscillatory growth of oxide layer on the {110} planes (**a**) Sequences of high-resolution TEM images of the growth on the Ag_2_O (110) surface under the electron beam irradiation with a dose rate of 9700 e·Å^−2^·s^−1^. (**b**) The growth length of the oxide layer as a function of time; insets show schematically the different growth stages of the oxide layer. Scale bar at 2 nm.

As mentioned above, the oxidation of Ag proceeds in layer-by-layer mode preferentially on {111} faces due to low surface free energy, while rhombic dodecahedra bounded by {110} faces is rarely available [25]. Surprisingly, we observed the addition of atom columns on the {110} planes at the atomic scale (Figure 4). The growth of the oxide layer started from the left corner and propagated laterally, which indicates that the growth of Ag_2_O is an adatom process on specific lattice planes.

Interestingly, competition was observed between the formation and the decomposition of the oxide layer during the growth of the oxide layers. As shown in Figure 4, during 76 s in the early stage, the atomic plane advanced along the surface and grew from 7.0 nm to 10.4 nm. However, the oxide layer retracted to 9.4 nm at 83.6 s, which indicates that the decomposition is significant at this moment. Then, the oxide layer resumed its growth and extended to 12.5 nm at 150.1 s. The oxide layer alternated between retraction and extension due to the competition between the decomposition and formation of the Ag_2_O. To further investigate the growth behavior of the growth of the oxide layer, the evolution of the length is plotted as a function of time in Figure 4b. Obviously, oscillatory growth frequently occurs during the entire oxidation process of Ag (see Figure S2 and Video S2 for more detail).

Several possible reasons can lead to the oscillation of growth. One possible explanation is that the as-formed oxide can be reduced by the electron beam [14,18]. Electron beam irradiation can provide both reducing species and ionized oxygen, and the concentration difference between reducing electrons and oxygen ions depends on the reaction of electron beam and the residual oxygen. Ionized oxygen could induce the formation of oxide, while reducing species decompose the oxide by reduction. The oscillatory growth of the Ag_2_O layer may originate from the competition of reducing electrons and oxygen ions. Moreover, the elastic sputtering can also induce the dissolution of oxide [26,27]. The Ag or O atoms can be knocked out from Ag_2_O by elastic sputtering under the electron irradiation at 300 keV. In summary, the role of electron beam irradiation in oscillatory growth involves two aspects: On the one hand, the incident electron beam provides ionic oxygen through decomposing O_2_ molecules, which could promote the growth of the oxide layer; on the other hand, electron beam can also induce the decomposition of oxide through the way of direct reduction or sputtering damage.

### 3.4. Growth Kinetics and Its Effect on Oxide Morphology

**Figure 5:**
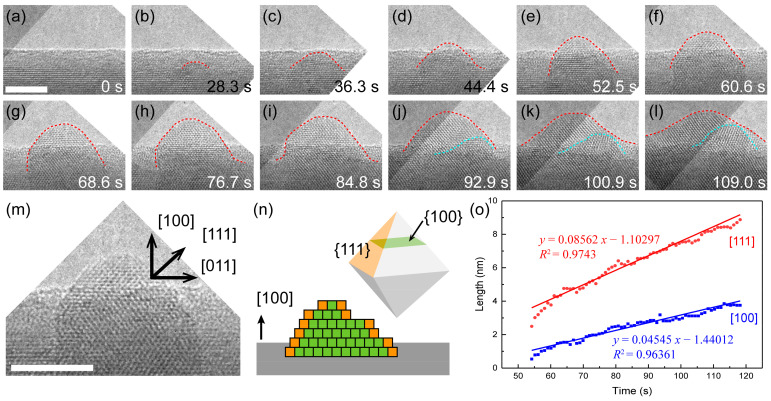
Growth kinetics of the low-index surface of Ag_2_O. (**a**–**l**) Time-resolved HRTEM images of atomic growth process of Ag_2_O nanocrystal. (**m**) HRTEM image showing the “hill-like” growth of Ag_2_O nanocrystal. (**n**) Scheme of the hill-like growth front. (**o**) Growth rate measured by the increment of oxide size vs. time. Dose rate at 10,600 e·Å^−2^·s^−1^. Scale bar at 2 nm.

As shown in Figure 5a–l, typical time-resolved HRTEM images (see Video S3 for more detail) show the growth process of Ag_2_O nanocrystal with a [100] zone-axis. The growth front of the oxide exhibited a hill-like curved surface (red dashed lines) composed of multiple atomic steps. At first stage of the growth (Figure 5b), a small crystallite nucleated at the subsurface of Ag nanowire. Then, more and more Ag atoms diffused to the top surface of the oxide and further reacted with the ionized O element to form Ag_2_O layers. The step edges were frequently observed in as-grown Ag_2_O crystals (Figure 5m and Figure S3), which indicates that the growth also proceeded in the mode of layer by layer on {111} faces.

The evolution of the length along [111] and [100] directions shows a linear growth rate along both directions (Figure 5o). The growth rate along the [111] direction is slightly faster than that of the [100] direction, indicating that the growth of Ag_2_O is selective growth of lattice planes. The difference in growth rate for different surfaces of Ag_2_O is contributed by the surface energy factor. The {111} planes of Ag_2_O has a higher packing density of surface atoms and thus a lower surface free energy, which indicates that {111} planes are preferred facets during the growth process and has a faster growth rate. In case there are more layers growing on {111} faces (Figure S3), the growth rate in the [111] direction would be larger, resulting in the development of an octahedron crystal (Figure 5n).

## 4. Discussion

The oxidation of Ag requires the presence of either atomic or ionized oxygen and can be described as:

2Ag + *i*O + (1 − *i*)O^−^ → Ag_2_O + (1 − *i*)e^−^ (1)

The atomic and ionized oxygen may be derived from the reaction with the residual O_2_ molecules in the chamber and those adsorbed on the Ag surface in several probable ways [18], such as

e^−^ + O_2_ → O^−^ + O (2)

e^−^ + O_2_ → 2O + e^−^ (3)

The surfactant and adsorbed water molecules adsorbed on the surface of Ag may also provide O species. Considering the pressure of 3 × 10^−5^ Pa in the column, the thickness of adsorbed water layer is about 0.7 nm, which corresponds to two double-layers of water molecules [28,29]. Under the electron beam irradiation, these water molecules adsorbed on the Ag surface could be excited into active oxidizing species and further react with Ag atoms.

Moreover, it is worth noting that no oxidation was found when the electron beam was only irradiated on the surroundings of the Ag nanowire. The oxidation process was only observed in hours after the specimen was inserted into the microscope. When a specimen holder is inserted into the chamber of the TEM, some air would inevitably enter the chamber, and the pressure could rise to ~3 × 10^−5^ Pa. Subsequently, the column vacuum can increase to the level of ~4 × 10^−9^ Pa with the assistance of a pump system and cold trap, and the oxidation reaction could no longer be observed then. These results indicate that the ionization of O_2_ molecules adsorbed [30,31,32,33,34,35] on the Ag surface plays a major role in the oxidation. In this case, the active species generated at the surface could react with the adjacent Ag atoms immediately. If the electron beam was illuminated on the surroundings of specimen, the active species generated in the chamber might be directly reduced by the incident electrons, and in this way few ionized O could diffuse and then react with the Ag nanowire.

The diffusion of Ag atoms is also a limiting factor that has a significant influence on the growth of the oxide. The layer-by-layer growth on {111} planes was observed in both Figure 2 and Figure 5. However, the morphologies of the two Ag_2_O crystals were different, which may be due to the different dominant orientation of them (see Figure S4 for more detail). The {111} faces in Figure 2 are parallel to the surface of the substrate, while the {111} planes in Figure 5 are tilted. With the continuous upward growth of the oxide layer on the {111} planes, it becomes more difficult for the replenishment of Ag atoms to reach the growth front, which limits the growth rate along the direction of the {111} faces (Figure 5). Limited by the diffusion of the Ag source, the growth of oxide is basically fixed along the surface of the substrate, even though the {111} faces are thermodynamically preferred. As a result, the growth rate along the surface of the Ag substrate is always larger than that of other directions.

During the growth of Ag_2_O, both thermodynamic and kinetics factors play important roles. It is believed that the growth of the oxide layer occurs on the surface that has a lower surface free energy via the mode of layer-by-layer growth. The {111} faces of Ag_2_O has a lower surface energy than the {110} faces due to the higher packing density of the surface atoms, so generally {111} faces in the Ag_2_O crystals are more common than {110} faces. However, under the electron beam irradiation, {110} planes with a higher surface energy could also maintain a stable state, which provides much convenience for us when recording the process that occurred on the {110} faces. Kinetic factors are considered to play significant roles during the process. The observed oscillatory growth on the {110} faces can also prove this. There is an energy difference between the {110} and {111} planes (with {111} planes being more stable than {110} ones), and less energy is required to decompose {110} faces [23]. The atoms can be more easily knocked out from {110} faces than that on {111} planes. However, further research is required to study the details of the competition between the formation and decomposition of the oxide layer.

In addition, the oxidation behavior of Ag could be controlled by several parameters. The oxidation reaction only occurs in the area irradiated by electron beam, and the oxidation area could be controlled by adjusting the irradiation location and the size of the electron beam. We tried to change the dose rate to control the oxide layer. When we focused the electron beam to increase the dose rate, the decomposition of oxide occurred. Thus, the oxidation that is controlled by electron beam irradiation could be used to fabricate a Ag-Ag_2_O metal–semiconductor contact and some functional devices. More detailed works are needed to study how to control the oxidation by adjusting electron beam dose rates and dwell times.

## 5. Conclusions

In summary, we directly visualized the oxidation process of Ag in real time at the atomic scale. The in situ HRTEM observations show that the oxidation process and the growth of oxide proceeds through the mode of layer by layer on specific lattice planes, and the outward surface diffusion of metal atoms plays an important role in the oxidation reaction. The electron beam irradiation, which provides the O sources by the ionization of adsorbed O_2_ molecules, is crucial for the oxidation process. The addition of columns of atoms on the {110} planes at the atomistic level was observed for the first time. Moreover, we also found the phenomenon of oscillatory growth of the top surface oxide layer, which was caused by the desorption of O from Ag_2_O or a direct reduction due to the electron beam. The growth kinetics and its influence on oxide morphology were discussed, and we demonstrated that a hill-like growth front with {111} planes is the preferred facet during the growth process as a result of the lower surface free energy. These findings not only throw light on a fundamental understanding of oxidation kinetics in Ag, but they also provide a new promising strategy for the precise fabrication of desired metal/oxide nanostructures.

## Data Availability

All the data supporting the findings of this study are available within this article and its Supplementary Materials files. All raw images and source data are available from the authors upon reasonable request.

## Supplementary Materials

The following are available online at https://www.mdpi.com/article/10.3390/nano11041021/s1, Figure S1. Characterization of the Ag nanowire. Figure S2. The oscillatory growth of oxide layer. Figure S3. The layer-by-layer growth occurred on {111} faces. Figure S4: HRTEM image of the Ag2O crystal Video S1. In situ oxidation on the Ag nanowire surface induced by electron beam irradiation. Video S2. The addition of columns of atoms on the (110) planes. Video S3. Growth kinetics of the low-index surface of Ag2O.
