# Supplementary material for: In Situ Atomic-Scale Observation of Silver Oxidation Triggered by Electron Beam Irradiation
Source: Nanomaterials (Basel). 2021 Apr 16;11(4):1021. doi: 10.3390/nano11041021 (PMC8073916; doi:10.3390/nano11041021)
Supplement: Supplementary file 1 [file nanomaterials-11-01021-s001.zip › nanomaterials-1142694-supplementary-final.pdf]

nanomaterials

MDPI

Supplementary Material

In Situ Atomic-Scale Observation of Silver Oxidation Triggered by Electron Beam Irradiation

Hui Zhang [1], Tao Xu [1,*], Yatong Zhu [1], Wen Wang [1], Hao Zhang [1], Dundong Yuan [1] and Litao Sun [1,2,*]

[1] SEU-FEI Nano-Pico Center, Key Laboratory of MEMS of Ministry of Education, School of Electronic Science and Engineering, Southeast University, Nanjing 210096, China; huiz@seu.edu.cn (Hui Zhang); 230189517@seu.edu.cn (Y.Z.); 230169400@seu.edu.cn (W.W.); 220191460@seu.edu.cn (Hao Zhang); 220191420@seu.edu.cn (D.Y.)

[2] Center for Advanced Materials and Manufacture, Southeast University–Monash University Joint Research Institute (Suzhou), Suzhou 215123, China

* Correspondence: xt@seu.edu.cn (T.X.); slt@seu.edu.cn (L.S.)

Citation: Zhang, H.; Xu, T.; Zhu, Y.; Wang, W.; Zhang, H.; Yuan, D.; Sun, L. In Situ Atomic-Scale Observation of Silver Oxidation Triggered by Electron Beam Irradiation. *Nanomaterials* **2021**, *11*, 1021. https://doi.org/10.3390/nano11041021

Academic Editors: Philip D. Rack; Giancarlo Rizza

Received: 25 February 2021
Accepted: 13 April 2021
Published: 16 April 2021

Publisher's Note: MDPI stays neutral with regard to jurisdictional claims in published maps and institutional affiliations.

Copyright: © 2021 by the authors. Submitted for possible open access publication under the terms and conditions of the Creative Commons Attribution (CC BY) license (http://creativecommons.org/licenses/by/4.0/).

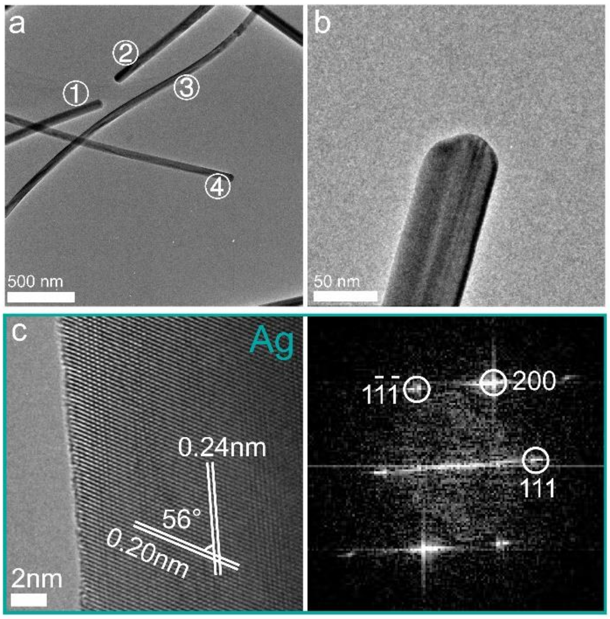

Figure S1. TEM images of Ag nanowires. (**a**) TEM image of 4 Ag nanowires, with different diameters of 72, 78, 68 and 65 nm (①, ②, ③ and ④, respectively). (**b**) The TEM image of an individual Ag nanowire, showing the twinned structure. (**c**) HRTEM image and the corresponding FFT pattern of Ag nanowire.

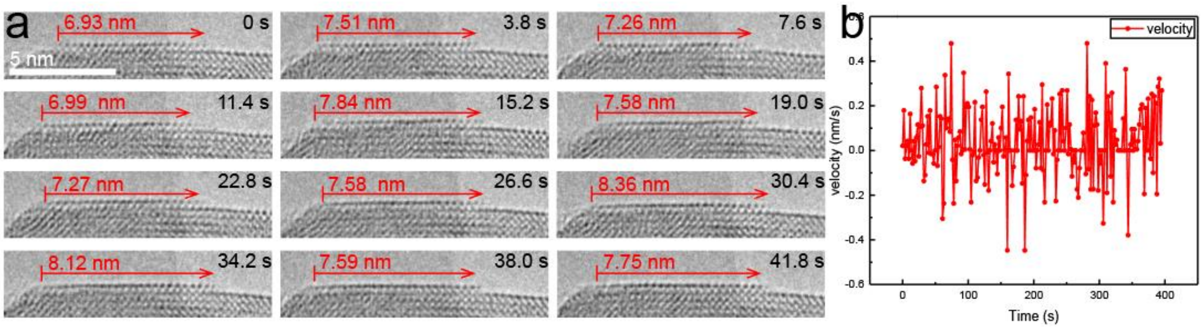

Figure S2. The oscillatory growth of oxide layer. (**a**) Sequences of high-resolution TEM images showing the oscillatory growth of a single Ag_2O layer. (**b**) Growth rate of the oxide layer as a function of time.

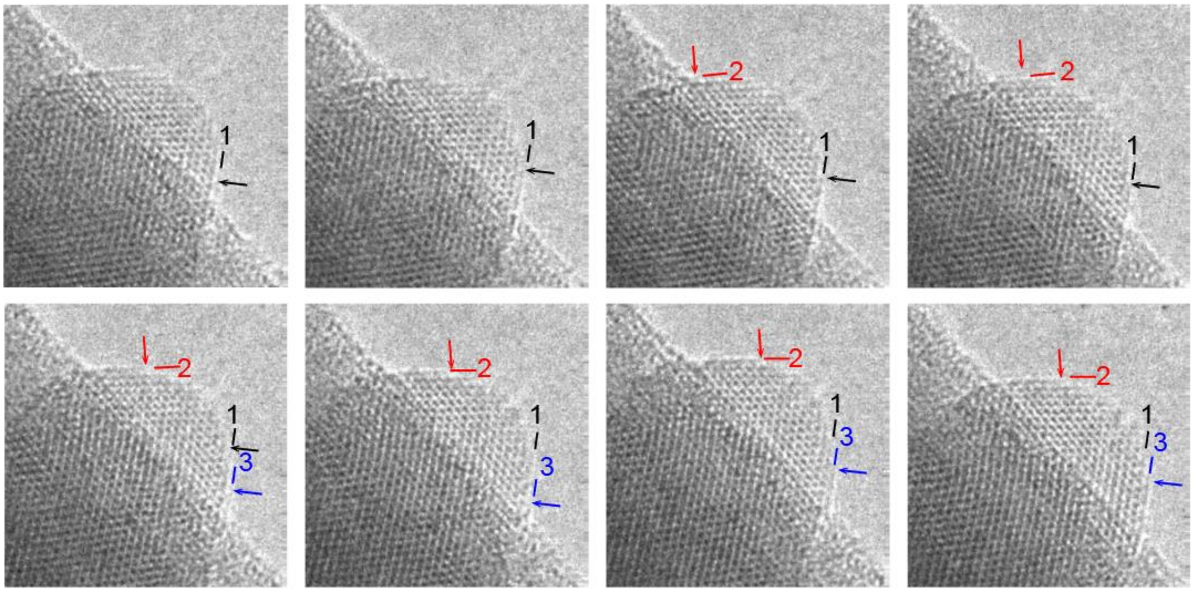

Figure S3. The layer-by-layer growth occurred on {111} faces. The arrows show the growth steps of three different oxide layer.

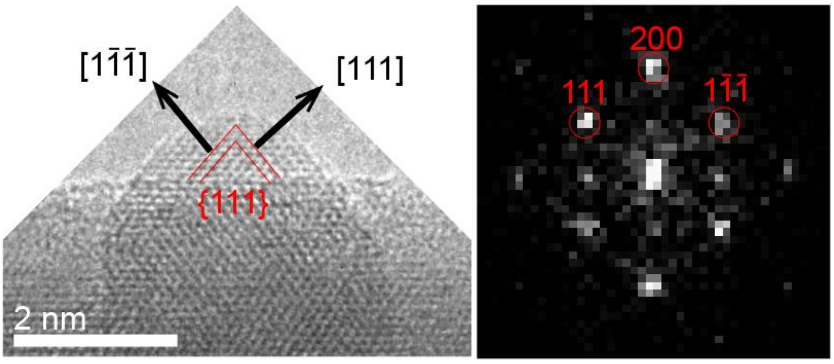

Figure S4. HRTEM image of the Ag_2O crystal shows the orientation relationship of {111} planes (including (111) and (1$\bar{1}\bar{1}$) faces).

Captions of Supplementary Videos

Video S1. In situ oxidation on the Ag nanowire surface induced by electron beam irradiation.

Video S2. The addition of columns of atoms on the (110) planes.

Video S3. Growth kinetics of the low-index surface of Ag_2O.
